# A phase I clinical trial of bavituximab and paclitaxel in patients with HER2 negative metastatic breast cancer

**DOI:** 10.1002/cam4.447

**Published:** 2015-03-31

**Authors:** Pavani Chalasani, Marilyn Marron, Denise Roe, Kathryn Clarke, Maria Iannone, Robert B Livingston, Joseph S Shan, Alison T Stopeck

**Affiliations:** 1 University of Arizona Cancer Center1515 N Campbell Ave, Tucson, Arizona, 85724; 2 Peregrine Pharmaceuticals, Inc.14282 Franklin Avenue, Tustin, California, 92780; 3 Stonybrook Medicine UniversityPO Box 1554, Stonybrook, New York, 11790

**Keywords:** Bavituximab, circulating tumor cells, metastatic breast cancer, microparticles, paclitaxel

## Abstract

Bavituximab is a chimeric monoclonal antibody that targets phosphatidylserine (PS). PS is externalized on cells in the tumor microenvironment when exposed to hypoxia and/or other physiological stressors. On attaching to PS, bavituximab is thought to promote antitumor immunity through its effects on PS receptors in monocytes, and myeloid-derived suppressor cells, as well as trigger antitumor effects by inducing an antibody-dependent cellular cytotoxicity on tumor-associated endothelial cells. We conducted a phase I clinical trial of bavituximab in combination with paclitaxel in patients with HER2-negative metastatic breast cancer. Patients were treated with weekly paclitaxel (80 mg/m^2^ for 3/4 weeks) and weekly bavituximab (3 mg/kg for 4/4 weeks). Correlative studies included the measurement of circulating microparticles, endothelial cells, and apoptotic tumor cells by flow cytometry. Fourteen patients with metastatic breast cancer were enrolled; all were evaluable for toxicity and 13 were evaluable for response. Treatment resulted in an overall response rate (RR) of 85% with a median progression-free survival (PFS) of 7.3 months. Bone pain, fatigue, headache, and neutropenia were the most common adverse effects. Infusion-related reactions were the most common adverse event related to bavituximab therapy. Correlative studies showed an increase in the PS-expressing apoptotic circulating tumor cells in response to bavituximab, but not with paclitaxel. No changes in the number of circulating endothelial cells or apoptotic endothelial cells were observed with therapy. Platelet and monocyte-derived microparticles decreased after initiation of bavituximab. Bavituximab in combination with paclitaxel is well tolerated for treatment of patients with metastatic breast cancer with promising results observed in terms of clinical RRs and PFS. The toxicity profile of bavituximab is notable for manageable infusion-related reactions with no evidence for increased thrombogenicity. Recent preclinical data suggest that bavituximab can also promote antitumor immune activity that should be explored in future clinical trials.

## Introduction

Metastatic breast cancer is the second leading cause of cancer-related deaths in women in the United States 1. Single-agent weekly paclitaxel has been proven to be well tolerated and effective, producing response rates (RRs) of 30–50% and median progression-free survival (PFS) rates of ∼6 months 2. For these reasons, weekly paclitaxel is one of the most common chemotherapeutic agents administered, either as a single agent or combined with targeted agents, to newly diagnosed metastatic breast cancer patients. The addition of the anti-Vascular Endothelial Growth Factor (VEGF) antibody, bevacizumab, to weekly paclitaxel resulted in improved RRs and an almost doubling of the median PFS in a large phase III trial 3. However, attempts to reproduce the results of E2100 using other chemotherapy agents or alternative antiangiogenic agents have been disappointing, suggesting weekly paclitaxel may be uniquely benefited when combined with bevacizumab 4,5. Many believe that the lack of a validated biomarker for predicting benefit to antiangiogenic agents has severely limited the optimal clinical development of these agents.

Bavituximab is an unconjugated, chimeric immunoglobulin G1 (IgG1) monoclonal antibody that targets phosphatidylserine (PS). Typically PS is restricted to the internal surface of the cell membrane. However, it is rapidly externalized on exposure to cell stressors often found in tumors, including hypoxia, chemotherapy, and radiation therapy 6,7. Thus, bavituximab binding and effects are relatively specific to the tumor microenvironment, where there is a high and constant degree of pathophysiological changes 8,9. In animal models, cytotoxic chemotherapy and radiation therapy have been shown to further induce PS exposure in tumors and associated blood vessels/stroma, thereby enhancing bavituximab binding and immune activation 10,11.

In addition to targeting PS on the tumor vasculature, bavituximab also alters the tumor immune milieu. Externalization of PS prevents antitumor immune and inflammatory reactions from occurring by inhibiting tumor-associated macrophages (TAM) and dendritic cell (DC) function 12. PS binds to TAM and DC through PS recognizing receptors like TIM3, TIM4, brain-specific angiogenesis inhibitor 1, stabilin-2, or receptor for advanced glycation end-products 13–16. Bound PS triggers production of transforming growth factor-*β* (TGF-*β*) and IL-10 and suppresses production of proinflammatory cytokines such as Tumor Necrosis Factor (TNF)^12^. By blocking the immunosuppressive signaling of PS, bavituximab has been shown to decrease TGF-*β* production in the tumor microenvironment, increase production of proinflammatory cytokines via Fc gamma receptor signaling, and induce antibody-dependent cellular cytotoxicity in murine tumor models 17. In preclinical models, bavituximab therapy has also been shown to induce monocytes and myeloid progenitor cells to differentiate into tumoricidal M1 macrophages, increase DC maturation and antigen presentation, and stimulate cytotoxic T-cell infiltration into tumors 12,18,19.

Bavituximab binding to PS is dependent on *β*_2_-glycoprotein I. Antiphospholipid autoantibodies, commonly known as lupus anticoagulants, similarly bind to *β*_2_-glycoprotein I in vivo; however, bavituximab binds to a different domain on the *β*_2_-glycoprotein I molecule than observed in lupus anticoagulants associated with thrombosis. This may explain why bavituximab therapy has not been associated with increased thrombosis in clinical trials to date. Recently a phase I trial was published indicating bavituximab can be safely administered to cancer patients with minimal to no toxicity at doses up to 3 mg/kg weekly 20. In order to advance the study of this agent in oncology patients, we performed this phase I study to further explore the safety and tolerability of bavituximab when combined with weekly paclitaxel in patients with HER2-negative metastatic breast cancer. Multiple biomarker assays were performed to determine the effects of bavituximab on the vasculature and on platelet or endothelial activity associated with angiogenesis or thrombosis.

## Patients and Methods

### Patient selection

Patients 18 years or older with metastatic HER2-negative breast cancer who had measurable disease by RECIST were eligible. The key inclusion criteria included Eastern Cooperative Oncology group (ECOG) performance status 0–2, adequate hematologic, renal, and hepatic function; prothrombin time (PT) within institutional normal limits (NL) and activated partial thromboplastin time (APTT) ≤1.5 upper limits of normal (ULN). Patients with a known history of bleeding diathesis or coagulopathy (e.g., von Willebrand Disease, hemophilia), any history of significant thromboembolic events (central venous catheter-related thrombosis were allowed if adequately treated), grade 2 or higher peripheral neuropathy, more than one prior chemotherapy regimen in the metastatic setting, symptomatic or clinically active brain metastases, serious nonhealing wound, chronic daily steroid use, HIV or hepatitis infection, cardiac arrhythmias requiring medical therapy and uncontrolled coronary artery disease were excluded.

### Study design

This was a single institution, phase I study to determine the safety and tolerability of bavituximab in combination with weekly paclitaxel in patients with metastatic HER2-negative breast cancer. This study was approved by the institutional review board and was conducted in accordance with US Food and Drug Administration Good Clinical Practice guidelines and the Health Insurance Portability and Accountability Act. All patients provided written informed consent.

**Figure 1:**
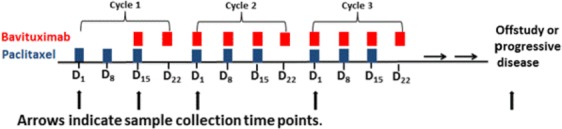
Schedule of paclitaxel and bavituximab treatment and collection of biomarkers (coagulation and angiogenic).

Patients were treated with weekly paclitaxel at 80 mg/m^2^ for three out of 4 weeks and weekly bavituximab 3 mg/kg for four out of 4 weeks. Each cycle was 28 days. For cycle 1 only, bavituximab was started on cycle 1 day 15 and then continued weekly to allow for analysis of changes in coagulation and angiogenic biomarkers in response to paclitaxel alone compared to paclitaxel combined with bavituximab. All blood for biomarker analyses were collected immediately prior to the patient receiving that day’s therapy. Thus, for the baseline time point, patients had not yet initiated therapy with either paclitaxel or bavituximab therapy; for the C1D15 time point, patient’s had received two doses of weekly paclitaxel alone, and for C2D1 and C3D1 time points patients were receiving combined paclitaxel and bavituximab treatment. The study schema is illustrated in Figure1. Patient assessments occurred weekly and included recording of adverse events, concomitant medications, physical findings, complete blood cell count with differential, serum chemistries, and liver function. APTT and D-dimer levels were obtained weekly during cycle 1 (day 1, 8, 15, 22) and day 1 of cycles 2 and 3. Tumor response assessment by Response Evaluation Criteria in Solid Tumors (RECIST version 1.1) was performed prior to study entry and at 12-week intervals (every three cycles) or as clinically indicated.

### Biomarkers

Blood was collected in sodium citrate (3.2% buffered sodium citrate solution) or Acid Citrate Dextrose (ACD) rose (trisodium citrate (22.0 g/L), citric acid (8.0 g/L) and dextrose (24.5 g/L)) tubes. Sodium citrate tubes were used for microparticle analysis and whole blood in ACD was used for apoptotic circulating endothelial cell (CEC), circulating tumor cell (CTC), and circulating endothelial progenitor (CEP) assays within 24 h of phlebotomy. For the apoptotic CTC analysis, whole blood was initially processed with Rosette Sep and Ficoll as described below. Blood for microparticle assays was processed within 30 min. Plasma collected in ACD tubes was frozen at −80°C and batched. Platelet poor plasma (PPP) was prepared within 2 h of venipuncture for use in microparticle analysis including platelet, endothelial cell, and monocyte cell microparticle (MP) assays. Fluorescence-activated cell sorting (FACS) was performed on a FACScan (BD Biosciences, Miami, FL) with Cell Quest Software for platelet activation assays, or an Accuri C6 Flow Cytometer (BD Biosciences) with CFlow Plus software for all other assays.

#### CECs and CEPs

CEC were measured by flow cytometry (Accuri C6) on whole blood using a RBC lysis, no wash method as previously described 21. Briefly, 100 *μ*L of ACD anticoagulated whole blood was incubated with the nuclear dye LDS751 (Molecular Probes, Eugene, Oregon), phycoerythrin (PE)-conjugated anti-CD31 monoclonal antibody (mAb) (BD PharMingen, Miami, FL), fluoresceinisothiocyanate (FITC)-conjugated anti-CD45 mAb (BD PharMingen), and allophycocyanin (APC)-conjugated anti-CD133-APC mAb (Miltenyi Biotec, Cologne, Germany). CECs were defined as the population of nucleated (LDS751 positive) cells that were negative for the pan-leukocyte marker CD45 and positive for endothelial cell marker CD31 (PECAM). CEPs were defined as the subpopulation of CECs which were also positive for progenitor marker CD133. The number of CEC or CEP/*μ*L was calculated as: (positive event count/bead count) × (total beads/100 *μ*L). Apoptotic CECs were defined by staining with the nonpermeable nuclear marker 7-aminoactinomycin D (7AAD) (BD PharMingen) to quantify late apoptotic CEC events. Apoptotic CECs were defined as platelet marker CD41a negative, leukocyte marker CD45 negative, CD31 (PECAM) positive, and 7AAD positive.

#### Apoptotic CTCs

CTCs were enriched from 2 mL of whole blood using RosetteSep Human Circulating Epithelial Tumor Cells Enrichment Cocktail (StemCell Technologies). Enriched CTCs collected in 2% fetal calf serum/1× D-PBS were stained with (APC)-conjugated anti-CD45 (BD PharMingen), epithelial cell marker (PE)-conjugated anti-EpCAM (epithelial cell adhesion marker) (BD PharMingen), 7AAD (BD PharMingen), and (FITC)-conjugated AnnexinV (Trevigen) in the presence of AnnexinV Binding Buffer (Trevigen, Gaithersburg, MD). CTCs were defined as the population of cells negative for leukocyte marker CD45 and positive for EpCAM. Events at least as large as granulocytes were gated to exclude smaller EpCAM-positive cellular fragments or microparticles from analysis. Apoptotic cells were defined by AnnexinV and 7AAD positivity with AnnexinV +7AAD− cells defining early apoptosis and AnnexinV +7AAD+ characterizing late apoptosis/necrosis.

#### Platelet activation

Platelet activation was measured by flow cytometry (FACScan) in whole blood within 30 min of venipuncture. Samples assayed for PAC-1 activation were stained with nuclear dye LDS751, (PE)-conjugated anti-CD31, and (FITC)-conjugated anti-PAC-1 (glycoprotein IIb/IIIa marker). Samples assayed for P-selectin activation were stained with LDS751, (FITC)-conjugated CD31, and (PE)-conjugated CD62p (P-selectin). Platelets were defined as LDS751 dim and CD31 platelet marker positive. Platelets were also treated with 10 *μ*mol/L of ADP as a positive control for P-selectin and 30 *μ*mol/L ADP for PAC-1 activation.

#### Microparticles

PPP was prepared within 30 min after blood collection by centrifugation of whole blood at 1500*g* for 10 min at room temperature (RT). PPP was subsequently centrifuged at 13,000*g* for 10 min (RT) generating platelet-free plasma (PFP). PFP samples were then diluted in 0.2 *μ*mol/L filtered 1X D-PBS (without calcium or magnesium) containing AnnexinV Binding Buffer (Trevigen) and stained with (FITC)-conjugated AnnexinV, peridinin–chlorophyll–protein complex (PerCP)-conjugated anti-CD45, and (APC)-conjugated CD41a. Microparticles were initially isolated by size using MegaMix fluorescent beads (Biocytex, Marseilles, France) and then cell of origin subtyped as platelet ([PMPs] AnnexinV positive, CD45 leukocyte marker negative, CD41a platelet marker positive, and CD62p positive if activated); Endothelial cell ([EMPs] AnnexinV positive, CD45 negative, CD41a negative, and CD31 [PECAM] positive); and monocyte ([MMPs] AnnexinV positive, CD41a negative, CD45 positive, and CD142 tissue factor positive).

### Statistical analysis

The sample size of 14 treated patients was determined to be sufficient to detect a ≥20% incidence of grade 3 or higher toxicities with a power of 0.9. Paired student *t*-tests were used to analyze biomarker results.

## Results

### Study population

A total of 14 patients were enrolled and completed at least one cycle of bavituximab and paclitaxel therapy. Table1 lists baseline patient characteristics. Eight patients had not received prior therapy for their metastatic disease, while four patients had progressed after endocrine therapy and two had progressed after receiving one prior chemotherapy regimen. All 14 patients were evaluable for safety. One patient had a catheter-related thrombosis prior to initiation of study treatments and continued on study without complications on therapeutic anticoagulation. One patient was eliminated from the study after three cycles after re-biopsy revealed her site of measurable disease progression was granulomatous disease. This patient was unevaluable for response, resulting in 13 patients evaluable for PFS.

**Table 1:** Baseline patient demographics

Characteristic	
Age at MBC diagnosis (yrs)	
Median	50
Range	43–81
Prior adjuvant treatment (*N*)	6
Prior neoadjuvant treatment (*N*)	2
ER+ (*N*)	7
Postmenopausal at MBC diagnosis (*N*)	7
Time to develop MBC diagnosis (yrs)	
Median	2
Range	0–10
Prior treatment for MBC (*N*)	
Hormonal therapy	4
Chemotherapy	2

MBC, metastatic breast cancer; *N*, number of patients; yrs, years.

### Safety

All 14 patients had at least one adverse event. Table2 lists the grade 3 or higher adverse events for all patients. The majority of the adverse events were grade 1 or 2 and attributed to paclitaxel treatment. Two patients discontinued treatment, one due to recurrent grade 3 infusion-related reaction and the other due to grade 3 hypertension in the setting of an infusion reaction. No patient had a thrombotic, bleeding, or grade 5 adverse events while on study.

**Table 2:** Overall summary of grade 3 or 4 adverse events

Adverse event	Grade 3	Grade 4	Grades 1–5
	*N* (%)	*N* (%)	*N* (%)
Back pain	2 (14)	0	7 (50)
Bone pain	2 (14)	0	6 (43)
Infusion reaction	1 (7)	0	2 (14)
Neutropenia	2 (14)	1 (7)	8 (57)
Peripheral neuropathy	2 (14)	0	11 (79)
Hypertension	1 (7)	0	1 (7)
Myalgia	1 (7)	0	5 (36)
Headache	1 (7)	0	6 (43)
Dyspnea	1 (7)	0	5 (36)
Diarrhea	2 (14)	0	7 (50)
Dehydration	1 (7)	0	1 (7)
Chest wall pain	1 (7)	0	1 (7)
Abdominal pain	1 (7)	0	3 (21)
Hyperglycemia	1 (7)	0	3 (21)
Syncope	1 (7)	0	1 (7)

*N*, number of patients, % of patients.

### Efficacy

**Figure 2:**
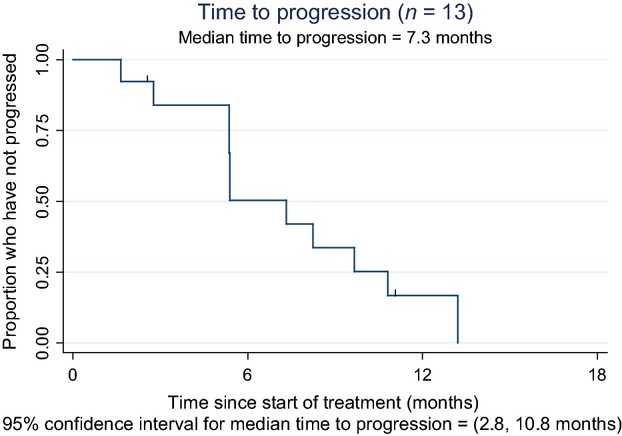
Kaplan–Meier curve showing progression-free survival for evaluable patients (*n* = 13). Median PFS = 7.3 months.

Thirteen patients were evaluable for response and PFS assessment. Median PFS for the combination of bavituximab with weekly paclitaxel was 7.3 months (2.8–10.8 months) (Fig.2).

**Figure 3:**
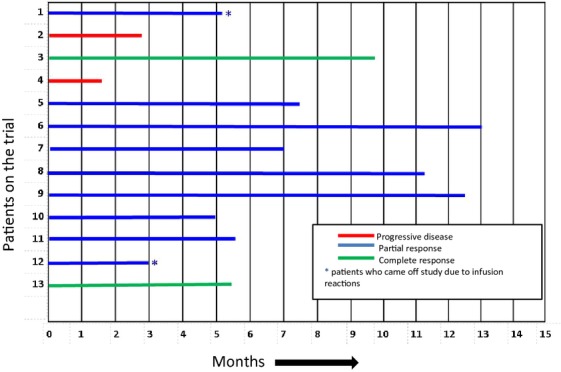
Duration of responses for evaluable patients (*n* = 13).

RR for the combination of bavituximab with weekly paclitaxel was 85% (11/13 patients) with two patients having complete responses (CR), nine with partial responses, and two with progressive disease (PD). Duration of responses ranged from 1.5 to 13 months (Fig.3).

### Biomarkers analysis

#### Apoptotic CECs, apoptotic CTCs, and total CEPs

**Figure 4:**
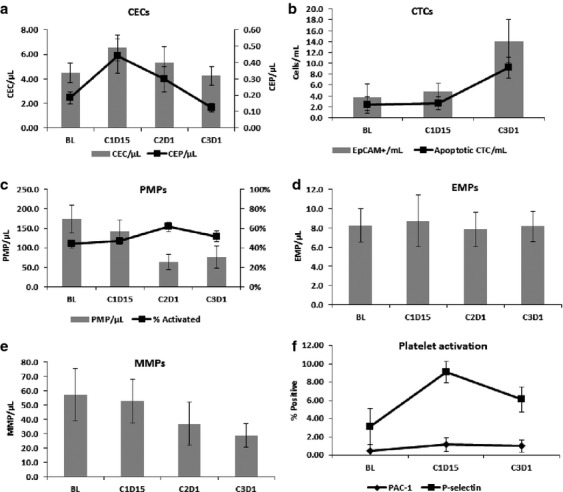
Biomarkers analysis. CEC and CEP cells did not change significantly (A), apoptotic circulating tumor cells increased over time (B), no significant change in platelet, endothelial, and monocyte microparticles (C–E) and platelet activation (F). CEC, circulating endothelia cells; CEP, circulating endothelial progenitor cells; CTC, circulating tumor cells; PMP, platelet microparticles; EMP, endothelial microparticles; MMP, monocyte microparticles.

Total CEC (Fig.4A) and apoptotic CEC values did not change over the course of therapy (*P* = 0.85). CEP cells’ values did not change significantly, but trended downward (*P* = 0.18) (Fig.4A). Total or apoptotic CTCs did not change after 2 weeks of therapy with paclitaxel; however, did increase after the addition of bavituximab to paclitaxel therapy (*P* = 0.05). The number of apoptotic cells also trended upward (Fig.4B). Both the number of early and late apoptotic EpCAM-positive cells increased significantly from baseline to cycle 3 (*P* = 0.004 and 0.009, respectively) and was primarily responsible for the increase in CTCs observed at cycle 3.

#### Platelet, endothelial cell, and monocyte microparticle generation

Platelet and monocyte-derived microparticles tended to decrease with the addition of bavituximab therapy with endothelial cell-derived microparticles remaining fairly constant over time (*P* = 0.96) (Fig.4C–E). Paclitaxel therapy alone did not significantly affect microparticle numbers. The number of measured PMP and MMP-derived microparticles at C2D1 after the initiation of bavituximab was significantly lower compared to baseline (*P* = 0.012 and 0.006, respectively) with the percentage of activated PMPs increasing (*P* = 0.038) as measured by P-selectin expression.

#### Coagulation parameters and platelet function

Platelet activation as measured by PAC-1 and P-selectin expression did not change significantly over the course of the study (Fig.4F). Other coagulation parameters like PT, activated thromboplastin time, d-dimer levels, and fibrinogen levels did not change over the course of therapy (data not shown).

## Discussion

Bavituximab is a novel chimeric IgG1 monoclonal antibody that targets PS externalized on tumor blood vessels inducing potential antitumor immunity and vascular disruption 12. This phase I study established the safety and tolerability of the combination of bavituximab and paclitaxel in patients with metastatic breast cancer. In addition, several pharmacodynamics biomarkers of coagulation and angiogenesis were performed in an effort to confirm the safety and mechanism of action of bavituximab in these patients.

The combination of weekly bavituximab with paclitaxel was well tolerated with the majority of adverse events being grade 1 or 2 and paclitaxel related. The observed RR of 85%, including two CR, and the 7.3 month median PFS are also encouraging and warrant further evaluation in an expanded trial. Two patients had grade 3 events related to bavituximab infusion reactions.

A unique aspect of this trial was the longitudinal quantification of cellular-derived microparticles, small membrane vesicles expressing high levels of PS. Microparticles are released from cells in response to activation, stress, or death (apoptotic microparticles) and have been found to be elevated in cancer patients 22,23. Cargo in microparticles includes various proteins (cytokines and growth factors), lipids, and nucleic acids (DNA, RNA, and miRNA) that can activate or be transferred to other cells (cell–to–cell communication) stimulating tumor progression and invasion, hypercoagulability, angiogenesis, drug resistance, host immunosuppression, and tumor survi-val 24–26. In patients with cancer, increased numbers of microparticles are generated and released into the blood stream on exposure to chemotherapy, hypoxia, and oxidative stress 23. Tumor, platelet, and monocyte-derived microparticles often express tissue factor and other procoagulants (i.e. P-selectin glycoprotein ligand-1) that can be activated on exposure to injured endothelium leading to coagulopathy and hypercoagulability 27. Elevated levels of TF-expressing microparticles have been associated with cancer-associated thrombosis in small series 28,29. Similarly, increased microparticle formation has been associated with thrombotic complications in patients with antiphospholipid antibody syndromes and microparticle quantification has been suggested as a potential biomarker for determining clinical significance of antiphospholipid antibodies in patients 30.

Because of the complexity and necessity of rapid (within 1 h of phlebotomy) evaluation, longitudinal quantification of microparticles in response to therapies has been rarely reported in cancer patients and to our knowledge, never reported in metastatic breast cancer patients. By initiating patients on therapy with weekly paclitaxel alone for two doses prior to infusing the first dose of bavituximab on day 15 of cycle 1, we were also able to distinguish changes in coagulation markers and microparticle formation in response to paclitaxel versus bavituximab combined with paclitaxel treatment. Our microparticle results are thus particularly intriguing as they suggest a rapid fall in platelet-derived and monocyte cell-derived microparticles in response to bavituximab, but not with initiation of paclitaxel chemotherapy. This may imply that by rapidly and specifically clearing prothombotic PS-expressing microparticles, bavituximab may actually decrease the risk of thrombosis in cancer patients. Other markers of platelet activation (PAC-1 and P-selectin) and thrombus formation (quantitative d-dimer levels) were also unaffected by bavituximab treatment further supporting our microparticle data. An increase in thrombotic events has also not been observed in any of the clinical trials involving bavituximab in cancer or hepatitis patients to date; however, the total number of patients treated with bavituximab remains small. Still, our results are encouraging and suggest increased thrombotic events may not be associated with this antibody despite its lupus anticoagulant-like activity in vitro 20.

An increase in PS-expressing apoptotic CTC values was also observed in patients after bavituximab therapy was initiated. Unfortunately, additional longitudinal measurements were not obtained and thus it is unclear if this increase in dead and fragmented CTC cells was secondary to increased tumor killing, a delayed immune response leading to increased tumor cell apoptosis, or damage to endothelial cells by the PS-targeted antibody resulting in a leaky tumor vasculature and thus more escape of tumor cell fragments into circulation. The CTC quantification also did not include a nuclear marker and thus we enlisted a gating strategy to ensure quantification of cells greater than ∼12 *μ*m in diameter. By also incorporating the use of apoptotic markers, annexin V and 7AAD, we were able to show that the increase in CTCs was primarily due to an increase in the release of early and late apoptotic cells into the circulation. Others have also suggested that the use of apoptotic markers on CTCs may be more predictive than simple quantification of intact CTCs in monitoring antitumor responses 31,32 and that CTC debris, fragments, and microparticles may be equally prognostic to intact CTCs in cancer patients 33.

Recent data suggest that macrophages and DC binding to PS can stimulate IL-10 and TGF-B-dependent immunosuppressive signals and help induce immune tolerance in the tumor microenvironment. By inhibiting PS interaction with immune cells and sending an alternate Fc-gamma receptor signal, bavituximab has been shown preclinically to foster antitumor immunity by potentiating M1 (immunostimulatory) TAM infiltrate 10,12,13. Such a delayed immune reaction may explain the increase in apoptotic CTCs observed; however, additional studies including analyses of immune infiltrating cells and the immune milieu of the tumor microenvironment are needed to establish this as a mechanism of action.

In conclusion, bavituximab in combination with weekly paclitaxel is a feasible regimen associated with an encouraging RR in patients with metastatic breast cancer. Our laboratory data suggest bavituximab may play a unique role in decreasing microparticle formation which may result in decreased hypercoagulability. Future studies are needed to explore bavituximab’s possible role in potentiating antitumor immunity and tumor-infiltrating macrophage responses.
